# The Effect of *Lactobacillus plantarum* 299v on Iron Status and Physical Performance in Female Iron-Deficient Athletes: A Randomized Controlled Trial

**DOI:** 10.3390/nu12051279

**Published:** 2020-04-30

**Authors:** Ulrika Axling, Gunilla Önning, Maile A. Combs, Alemtsehay Bogale, Magnus Högström, Michael Svensson

**Affiliations:** 1 Probi AB, 223 70 Lund, Sweden; gunilla.onning@probi.com; 2 Biomedical Nutrition, Pure and Applied Biochemistry, Center for Applied Life Sciences, Lund University, 221 00 Lund, Sweden; 3 Nutrition and Scientific Affairs Department, The Nature’s Bounty Co., Ronkonkoma, NY 11779, USA; mcombs@nbty.com (M.A.C.); abogalewot@nbty.com (A.B.); 4 Sports Medicine Umeå AB and Orthopedics, Department of Surgical and Perioperative Sciences, Umeå University, 901 87 Umeå, Sweden; magnus.hogstrom@sportsmedumea.se; 5 Section of Sports Medicine, Department of Community Medicine and Rehabilitation, Umeå University, 901 87 Umeå, Sweden

**Keywords:** *Lactobacillus plantarum* 299v, DSM 9843, probiotics, athletes, iron-deficiency, physical performance, exercise, ferritin, vigor

## Abstract

Iron is an essential micronutrient for oxygen transport and mitochondrial metabolism and is critical for physical performance. Compromised iron stores are more commonly found among athletes, and females are especially at risk. Iron deficiency is generally treated using oral iron supplements. However, only a small proportion of ingested iron is absorbed, necessitating higher intakes, which may result in adverse side effects, reduced compliance, and inefficient repletion of iron stores. The probiotic strain *Lactobacillus plantarum* 299v (Lp299v) significantly increases intestinal iron absorption in meal studies. The present study was conducted to explore the effects of 20 mg of iron with or without Lp299v on iron status, mood state, and physical performance. Fifty-three healthy non-anemic female athletes with low iron stores (ferritin < 30 μg/L) were randomized, and 39 completed the study. Intake of Lp299v with iron for four weeks increased ferritin levels more than iron alone (13.6 vs. 8.2 µg/L), but the difference between the groups was not significant (*p* = 0.056). The mean reticulocyte hemoglobin content increased after intake of Lp299v compared to control (1.5 vs. 0.82 pg) after 12 weeks, but the difference between the group was not significant (*p* = 0.083). The Profile of Mood States (POMS) questionnaire showed increased vigor with Lp299v vs. iron alone after 12 weeks (3.5 vs. 0.1, *p* = 0.015). No conclusive effects on physical performance were observed. In conclusion, Lp299v, together with 20 mg of iron, could result in a more substantial and rapid improvement in iron status and improved vigor compared to 20 mg of iron alone. A larger clinical trial is needed to further explore these findings as well as the impact of Lp299v on physical performance.

## 1. Introduction

Iron deficiency (ID) is a global health problem. Iron deficiency refers to the reduction of iron stores and is the most common cause of anemia [1,2]. Among else, iron is crucial for oxygen transport, mitochondrial energy production, and cellular immune responses [3]. Compromised iron stores, even without iron-deficiency anemia, have been shown to negatively affect physical performance and adaptation to training [4,5,6]. Low iron stores are more commonly found among athletes than in non-athletes, and female athletes are especially at risk [7,8,9,10,11,12,13,14]. The prevalence of ID in females involved in competitive sports has been reported to range from 10 to 38% [10,11,15,16,17] and to vary between training seasons [7,10,18].

The increased risk of ID and iron deficiency anemia in athletes is thought to be due to factors, such as foot-strike induced hemolysis, insufficient dietary intake, increased iron losses, and suppressed intestinal iron absorption caused by inflammation [19,20,21,22]. Ferritin is the cellular storage protein for iron, and circulating levels are generally considered reflective of total body iron stores [23]. Ferritin seems to adequately identify most of the ID in a majority of athletes [10,11,15,16,17] and is, therefore, a useful marker of iron status.

Some studies have demonstrated the beneficial effects of iron supplementation on physical performance in women of reproductive age [24], but it has also been questioned whether iron supplementation to iron-deficient non-anemic subjects improves physical performance [5]. Iron deficiency is generally treated using oral iron supplements. More than 50% of female athletes have been shown to use dietary supplements, and those containing iron appear to be among the most popular [25]. However, a large proportion of the ingested iron typically remains in the gut, necessitating higher intakes, which may result in adverse side effects, reduced compliance, and limited ability to efficiently replete iron stores [26,27]. In addition, heavy training per se can be stressful for the intestinal tract, resulting in gastrointestinal discomfort [28,29]. Furthermore, emerging data suggest that the non-absorbed iron could be harmful through modifications of the gut microbiota, increasing the concentration of intestinal pathogens [30]. Therefore, increasing the absorption of iron could be a strategy for improving iron status and avoid the use of traditional high-dose iron supplements and thereby adverse side effects. It has previously been shown that the probiotic strain *Lactobacillus plantarum* 299v (Lp299v, LP299V^®^) significantly increases iron absorption in meal studies [31,32,33]. The use of probiotic supplements for optimizing health, performance, and recovery in athletes has been recently reviewed by the International Society of Sports Nutrition [34].

The aim of this exploratory study was to evaluate the effects of a daily intake of 10^10^ colony forming units (CFU) of Lp299v together with 20 mg of iron per day compared to 20 mg of iron alone on iron status, mood status, and parameters relating to physical performance. The study was conducted in a population of healthy, non-anemic, female athletes with low iron stores. 

## 2. Materials and Methods

### 2.1. Study Design

The study was a randomized, double-blind, placebo-controlled, parallel study with the objective to evaluate the effect of daily intake over 12 weeks of *Lactobacillus plantarum* 299v (LP299V^®^; 10^10^ CFU) and 20 mg of iron (ferrous-fumarate; LpFe) compared to 20 mg of iron alone (CtrlFe) on iron status and physical performance assessed by ergometer cycling tests. The study was comprised of one screening visit and four study visits at baseline, weeks 4, 8, and 12. The study was carried out at one study site in Sweden between Aug 2017 and May 2018. All subjects gave their informed consent before participating in the study. Participation was voluntary and could be discontinued at any time without explanation. The study was conducted in accordance with the Declaration of Helsinki, and the protocol was approved by the ethics committee in Umeå, Sweden. The trial was registered at ClinicalTrials.gov prior to the study start (NCT03259997). The subjects were randomized at a ratio of 1:1 to receive Lp299v product or control. The generation of the randomization list was delegated to an independent biostatistician. Stratification was performed based on plasma ferritin levels (≤20 µg/L or >20 µg/L) at baseline in order to reduce the risk of differences in baseline ferritin between the groups. The study subjects were enrolled and assigned to the intervention by the investigator. The allocation of study products was not disclosed to the investigator, other staff, or sponsor until clean file was declared. The blinding was maintained throughout the study.

### 2.2. Study Population

In the present study, 365 athletes (231 females and 134 males) were screened in order to find eligible subjects; healthy non-anemic (hemoglobin ≥ 120 g/L for females, ≥130 g/L for males) males and females aged 16–40 years old, with low iron stores (plasma ferritin < 30 μg/L) and high-sensitive C-reactive protein (hCRP) ≤ 5 mg/L, engaged in competitive sports and regularly training ≥5 h/week. Exclusion criteria were the use of probiotic products, iron supplementation, or ascorbic acid supplementation during the last 4 weeks prior to the start of the intervention, plans for substantial changes in diet and/or training (as judged by the investigator), and pregnancy or plans for pregnancy within the following 20 weeks. Subjects were instructed not to consume probiotic products, additional iron supplements, or ascorbic acid supplements during the study. Subjects were also asked to avoid changes in the intake of dietary supplements and to avoid major changes in dietary habits. The subjects could not donate or receive blood during the study.

### 2.3. Study Product

The study product was provided as capsules. The Lp299v product (LpFe) contained freeze-dried probiotic *Lactobacillus plantarum* 299v at a concentration of 10^10^ CFU/capsule, 20 mg of iron (ferrous-fumarate), maize starch (bulking agent), maltodextrin (bulking agent), cellulose derivatives (coating of iron), and magnesium stearate (processing aid/anti-caking). The capsule consisted of hydroxypropyl methylcellulose and titanium dioxide (white color). The control product (CtrlFe) contained all ingredients except Lp299v. Capsules were of identical appearance. The subjects were instructed to consume the study product, one capsule per day, in connection to the main meal of the day. Compliance was assessed by counting of returned study products and through study diaries. 

### 2.4. Recruitment and Screening

Subjects were recruited through local sports clubs and regional sports schools. Hemoglobin (Hb), plasma ferritin, and hCRP were analyzed in order to identify eligible subjects.

### 2.5. Demographics and Anthropometrics

Demographic information (date of birth and gender) was recorded at the screening visit. Height and weight were recorded at the first study visit and were used to calculate body mass index (BMI, kg/m^2^).

### 2.6. Blood Sampling and Analyses

Blood samples were drawn via a polyethylene catheter inserted into a superficial forearm vein at each study visit. The blood samples were analyzed for hemoglobin, erythrocyte volume fraction (EVF), plasma iron, total iron-binding capacity (TIBC), transferrin saturation, plasma ferritin (P-Ferritin), reticulocytes, mean reticulocyte hemoglobin content (Ret-Hb), hCRP, hepcidin, soluble transferrin receptor (sTfR), and hepcidin at certified university hospital laboratories using standardized and validated procedures. TNFα, IL-6, and IL-1β were analyzed using the mesoscale platform according to the manufacturer’s instructions.

### 2.7. Ergometer Cycling Test

In order to standardize the ergometer cycling test as much as possible, the subjects were instructed to prepare in the same way 24 h before each test in terms of food intake and rest. Before the start of the test, adherence to the standardized preparations was confirmed. Subjects were asked to fill out a general health status questionnaire before blood sampling and ergometer cycle test at each of the four study visits, and the study personnel ensured that the subjects were in good health to safely carry out the test. Relevant current diseases and medications were recorded.

Subjects performed a standardized ergometer cycling test that was divided into two parts; (1) 30 min of submaximal ergometer cycling starting with 10 min of warm-up at 50 watts (W), followed by 5 min each at 75 W, 100 W, 125 W, and 150 W, respectively, then 5 min of recovery before the start of (2) maximal ergometer cycling until time of exhaustion. The starting workload at the maximal test was individually set depending on the individual’s lactate level and the self-rated perceived exertion with the Borg 6–20 RPE scale at the end of the last submaximal workload. The workload increased with 20–25 W each minute until time of exhaustion at the maximal test. The measurement of oxygen uptake (VO_2_) and carbon dioxide production (VCO_2_) was done during the submaximal, as well as the maximal ergometer cycling test through indirect calorimetry (AMIS Sport, Innovision Aps, Glamsbjerg, Denmark). The maximal oxygen uptake (VO_2_max) was also determined, as the highest mean of 60 consecutive seconds, during the maximal ergometer cycling. Venous blood was drawn via the polyethylene catheter at the end of each submaximal workload, as well as 3 min after completion of the maximal ergometer cycling test for analyses of lactate using a Biosen C line system (EKF Diagnostics GmbH, Barleben, Germany). Heart rate was measured during submaximal and maximal workloads. A Monark ergometer cycle (Monark Ergomedic 839E, Vansbro, Sweden) and a Polar heart rate monitor with a Polar H7 Sensor (Polar Electro Oy, Kempele, Finland) were used for the test. Self-rated perceived exertion, according to the Borg 6–20 RPE scale [35], was registered at the end of each submaximal workload and maximal workload. Work efficiency, i.e., energy consumption in relation to each workload during ergometer cycling, was calculated from VO_2_ and VCO_2_ measured via indirect calorimetry at steady-state during the last minute of each submaximal workload, according to Jeukendrup et al. [36].

### 2.8. Profile of Mood States (POMS)

The Profile of Mood States (POMS) questionnaire, according to McNair et al. [37], was used to assess the Profile of Mood States at each study visit and was carried out prior to the ergometer cycling test. The test includes 65 mood-related adjectives, which are rated on a 5-point Likert scale, ranging from 0 (not at all) to 4 (extremely) in response to the question “How are you feeling right now”. Six sub-scores can be derived from the test (vigor, tension, depression, anger, fatigue, and confusion), as well as a total mood score. The results are presented as T scores.

### 2.9. Safety and Gastrointestinal (GI) Well-Being

Safety was assessed by the recording of adverse events at each study visit, both via discussion with the subject as well as through interpretation of the results from the general health questionnaire and a study diary. Gastrointestinal (GI) wellbeing was evaluated, according to Guyonnet et al. [38]. The subjects were asked to rate their wellbeing/comfort, using a 3-point Likert scale (improved, unchanged, worse) by answering the following question: “How do you consider your GI wellbeing (intestinal transit, stool frequency and consistency, abdominal pain/discomfort, bloating, flatulence/passage of gas, borborygmi/rumbling stomach) in the past 7 days, compared to the period before beginning the consumption of the study product?”. The subject rated their GI wellbeing after 4, 8, and 12 weeks of intake of the study product.

### 2.10. Statistical Methods

In this exploratory study, we aimed to include a total of 40 eligible subjects. The Wilcoxon signed-rank test was used when evaluating change over time within each group, and the Wilcoxon rank-sum test was used when evaluating differences between groups. Fisher’s exact test was used for evaluation of the prevalence of upper respiratory tract infections (URTI) between the two groups. All reported *p*-values were two-sided and nominal, i.e., not adjusted for multiple testing. Statistical analyses were carried out by an independent biostatistician using Microsoft Excel 2016 (Microsoft Office) and StatXact version 11.1.0 (Cytel Software Corporation, Cambridge, MA, USA). The main analyses set consisted of all subjects with no major protocol deviations and compliance above 80% for each four-week period (per-protocol population). If nothing else was stated, the reported data were from the per-protocol population.

## 3. Results

### 3.1. Subject Disposition and Baseline Characteristics

**Figure 1:**
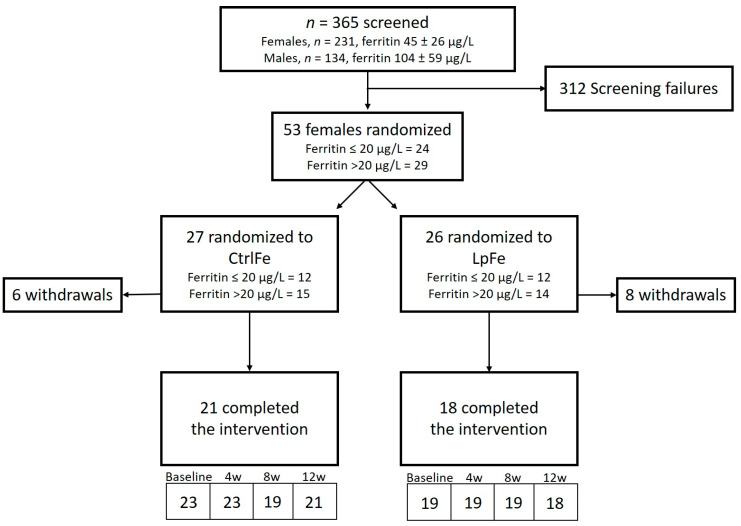
Study flow chart. CtrlFe denotes the group receiving 20 mg of iron alone; LpFe denotes the group receiving 20 mg of iron together with *Lactobacillus plantarum* 299v. The numbers in the lower boxes correspond to the number of subjects at each study visit.

In total, 365 subjects were screened for eligibility. Fifty-three (53) females fulfilled all inclusion criteria and none of the exclusion criteria and were randomized. During the intervention, 14 subjects were excluded or withdrew their consent. The subject disposition is presented in Figure 1. No eligible male subjects were found in the screening process, and, therefore, only female subjects were included in the study. There were no differences in average training volume, age, body weight, or BMI between the groups at the start of the intervention. The mean age was 22 years, and the mean average training time per week prior to the start of the study was 8 h per week (Table 1).

### 3.2. Iron Status

The mean ferritin levels for all the screened subjects were 104 µg/L for males and 45 µg/L for females. Out of the 231 females, 6.5% had ferritin levels below 15 µg/L, and 24% had ferritin levels between 15 and 29 µg/L. The mean plasma ferritin level for the study population was just below 20 µg/L at baseline, indicating low iron stores among the participants at the start of the study, while the mean hemoglobin level was 130 g/L (Table 2). Baseline iron status was comparable between the two groups.

**Figure 2:**
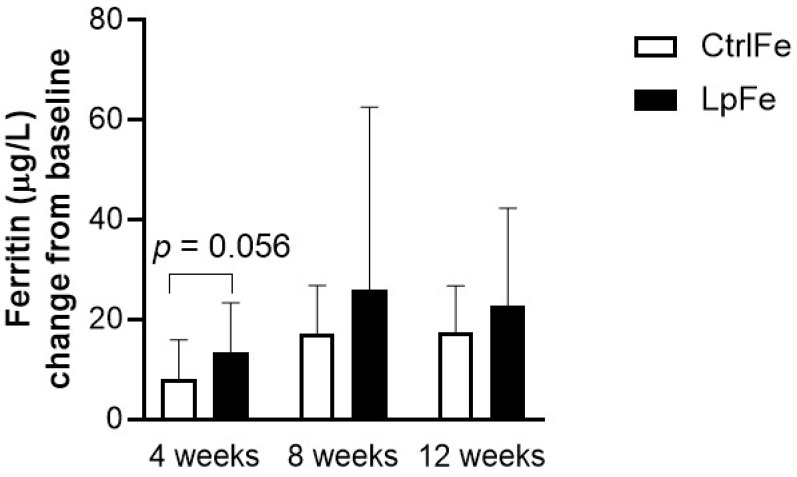
Change from baseline in plasma ferritin at 4, 8, and 12 weeks of intervention. Data are presented as mean values with SD. *n* = 16 for LpFe and 22 for CtrlFe at week 4, *n* = 16 for LpFe and 17 for CtrlFe at week 8, and *n* = 15 for LpFe and 17 for CtrlFe at week 12.

The ferritin levels increased significantly in both groups, from 19.5 to 43.0 µg/L in the LpFe group (*p* < 0.001) and from 19.8 to 37.8 µg/L in the CtrlFe group (*p* < 0.001) over the whole study period. No significant difference in change from baseline could be detected between the two groups (13.6 µg/L (95% CI 8.3–18.8) vs. 8.2 µg/L (4.8–11.6), mean change from baseline to week 4, *p* = 0.056, Figure 2). There were no differences in the prevalence of ID (ferritin < 20 µg/L) between the two groups over the study period (10% in the LpFe and 9% in the CtrlFe at week 4). After four weeks, 7 out of 17 (41%) in the LpFe group and 7 out of 22 (32%) in the CtrlFe group reached a ferritin level of 30 µg/L or above, and 29% in the LpFe group and 9% in the CtrlFe reached a ferritin level of 40 µg/L or above.

**Figure 3:**
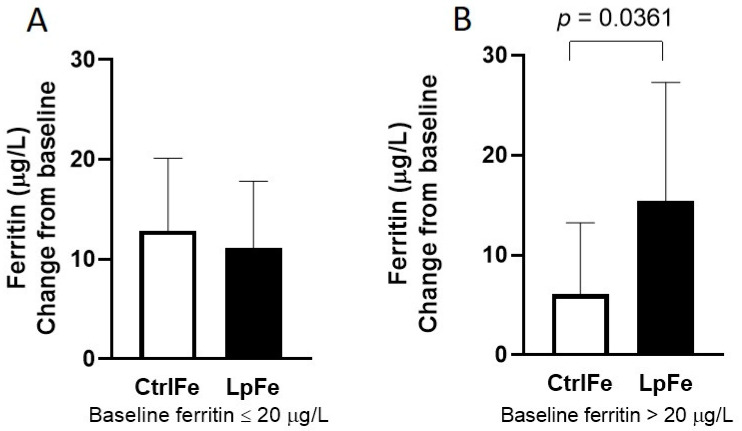
Change from baseline in plasma ferritin after 4 weeks of intervention in the subgroup of subjects with a ferritin level below (**A**) and above 20 µg/L at baseline (**B**). Data are presented as mean values with SD. *n* = 7 for CtrlFe and 7 for LpFe in A, and *n* = 15 for CtrlFe and 9 for LpFe in B.

When evaluating subgroups based on their baseline plasma ferritin levels (below or above 20 µg/L), the LpFe subgroup with a ferritin level above 20 µg/L showed a significantly higher increase (15.4 µg/L (95% CI 6.3–24.6) vs. 6.1 (2.1–10.0) µg/L, *p* = 0.0361, Figure 3) in ferritin after 4 weeks of intake compared to CtrlFe, while the increase in ferritin in the two subgroups with a ferritin level below 20 µg/L did not differ (12.9 µg/L (95% CI 6.1–19.6) vs. 11.1 (5.0–17.3) µg/L, *p* = 0.5437) from each other. No differences were detected for the other time points.

The hemoglobin content of the reticulocytes tended to increase in the LpFe group (1.5 pg/L (95% CI 0.8–2.2) vs. 0.82 (0.3–1.3) pg/L, change from baseline to week 12, *p* = 0.0834) compared to CtrlFe. The CtrlFe displayed an increase in total plasma iron over the first four weeks compared to the LpFe group (5.6 vs. −1.6, *p* = 0.0411, Table 3), but no difference was detected over the whole study (2.6 vs. −0.27, *p* = 0.4373 for change from baseline to 12 weeks). There were no significant changes over time or between groups in hemoglobin, plasma transferrin, plasma transferrin saturation, EVF, blood reticulocytes, sTfR, hepcidin, and hCRP levels (Table 3).

### 3.3. Inflammatory Parameters

The concentrations of circulating CRP, IL-6, IL-1β, and TNFα were low, and there were no detectable differences between the two groups at any of the study visits.

### 3.4. Physical Performance

Based on the results for heart rate, VO_2_, VCO_2_, and ventilation during submaximal ergometer cycling, no evident difference in cardiovascular-respiratory response or work efficiency was found between the baseline and after 4, 8, and 12 weeks of supplementation, respectively, or between groups. Besides, no change in perceived exertion was detected over the intervention period in any group during submaximal ergometer cycling. The blood lactate levels during the submaximal ergometer cycling test displayed incongruent results without any clear change within or between the groups, from baseline and after 4, 8, and 12 weeks of intake, respectively. Despite significantly higher VO_2_max following 4 (3.1 vs. 2.9, *p* = 0.0472), 8 (3.1 vs. 2.8, *p* = 0.0019), and 12 (3.0 vs. 2.7, *p* = 0.0447) weeks with the intake of LpFe, compared to intake of CtrlFe (Table 4), no significant change in VO_2_max or endurance (time to exhaustion) was detected between the two groups. The LpFe group displayed higher blood lactate levels three minutes post maximal ergometer cycling after 4 (10.8 vs. 8.3, *p* = 0.0139) and 8 (10.8 vs. 8.3, *p* = 0.0343) weeks of supplementation compared to CtrlFe, together with a tendency towards a higher increase in blood lactate levels compared to CtrlFe after the first four weeks (0.47 vs. −0.72, *p* = 0.0562), but no detectable difference was observed over the whole 12 weeks (0.02 vs. −1.05, *p* = 0.3346,). It should be noted that large differences between subjects were found in physical performance and that fewer subjects in both groups performed the cycling test at 8 and 12 weeks than at baseline and 4 weeks due to colds/URTIs), e.g., the change from baseline to week 4 included a larger number of comparisons than the change from baseline to weeks 8 or 12.

### 3.5. Profile of Mood Score

There was a significant increase in the “vigor” T-score of the POMS questionnaire after 12 weeks in the LpFe group (mean T-score of 58 (95% CI 55–61) to 61 (CI 58–65), *p* = 0.044), but no differences were detected in the CtrlFe group (mean T-score of 58 (95% CI 55–61) to 59 (56–63), *p* = 0.981). This change from baseline to week 12 was significantly different for the LpFe group compared to the CtrlFe group (3.5 (SD 6.3; 95% CI 0–7) vs. 0.13 (3.5; −2–2), *p* = 0.015). No other differences between the groups could be detected, neither for the total mood profile nor for any of the other sub-scores.

### 3.6. Safety and GI Well-Being

A total of 51 adverse events were reported—32 by subjects in the CtrlFe group and 19 by subjects in the LpFe group. There were two withdrawals due to adverse events (prolonged URTIs between randomization and the baseline visit), both subjects in the LpFe group. Eighteen subjects in the CtrlFe and 15 subjects in the LpFe group reported at least one adverse event. Upper respiratory tract infections during the study were reported in both the LpFe and CtrlFe groups. The incidence of URTIs in the LpFe group was lower, 5 of 19 (26%), compared to the CtrlFe group, 14 of 23 (61%; *p* = 0.0332).

## 4. Discussion

In the present study, the effect of the probiotic strain *Lactobacillus plantarum* 299v^®^ (Lp299v, LP299V^®^) on iron status, physical performance, and mood was evaluated in non-anemic female athletes with low iron stores receiving a daily supplement of 20 mg of iron for 12 weeks.

Over the 12-week study, both groups increased their iron status, displayed by a 70% increase in plasma ferritin after intake of Lp299v with 20 mg of iron as compared to 42% after 20 mg iron alone, but the difference between groups did not reach statistical significance. However, the LpFe group displayed a tendency towards higher ferritin levels after the first 4 weeks compared to control. This finding is interesting since it is important for iron-deficient athletes to quickly restore iron status without adverse events, which often occur with higher doses of supplemental iron [26]. The LpFe subgroups with ferritin levels above 20 µg/L at baseline showed a significantly higher increase in ferritin after 4 weeks of intake compared to control, while the subgroups with ferritin levels below 20 µg/L did not differ from each other. Thus, it appears that the overall increase in ferritin with LpFe is driven by subjects with better baseline iron status. Greater improvement in iron status with LpFe was further indicated by a trend towards increased mean hemoglobin content in the reticulocytes, after 12 weeks of intake. The reticulocyte hemoglobin content is an indicator of cellular iron availability in response to iron supplementation and an early marker of improved erythropoiesis [39,40].

It has previously been shown that the intake of Lp299v improves intestinal iron absorption in meal studies [31,32,33]. In addition, the intake of Lp299v has been shown to increase the hemoglobin and hematocrit values in anemic rats [41]. The beneficial effect of Lp299v on iron absorption has also been recently examined in a systematic review and meta-analysis [42]. The underlying mechanisms are not fully known, but Lp299v has been shown to increase the amount of ferric iron in in vitro digested meals and drinks. This, in combination with the ability of Lp299v to increase levels of a ferric reductase (duodenal cytochrome B, DcytB) in human intestinal cells (Caco-2/HT29 MTX cells), may explain the positive effect on iron absorption [43]. In the current study, we showed that the effect of Lp299v on intestinal iron absorption translated into an improved iron status in a non-anemic, iron-deficient population when administered in combination with a low dose of iron. Although iron absorption per se was not analyzed in this study, it was theorized that increased iron absorption is at least one of the underlying mechanisms of the improved iron status.

It has been shown that in inflammatory states, a systemic elevation of pro-inflammatory cytokines, such as IL-6 and TNFα, inhibits intestinal absorption of iron through elevation of hepcidin [44]. Cytokines may also suppress iron uptake through a hepcidin-independent pathway [45]. Interestingly, exhaustive exercise has been shown to increase IL-6 and hepcidin levels [46], which may be one among several factors that cause iron deficiency in athletes following intense periods of training. It is known that intake of Lp299v suppresses inflammatory parameters [47,48,49], and, therefore, we posited that this could lead to a change in hepcidin expression and, thus, iron absorption. However, in the present study, we were unable to detect any differences over time between the groups for selected inflammatory cytokines or for hepcidin, partially due to the very low cytokine levels. Interestingly, the increase in ferritin after intake of Lp229v was higher in the subgroup of subjects with greater iron status. It is possible that the athletes with the lowest ferritin status had the highest levels of pro-inflammatory cytokines that stimulated an increase in hepcidin level and, thus, increased the inhibition of iron uptake. However, we did not detect any differences in hepcidin, CRP, or IL-6 between the small subgroups in our study. A larger study would be needed to further explore this and investigate inflammatory markers and hepcidin right after an exercise bout when the degree of inflammation is most likely to be higher.

In this study, total plasma iron increased over the first four weeks in the control group compared to the LpFe group. Plasma iron by itself is not normally considered when examining iron status; instead, total iron is used together with transferrin concentrations (i.e., total iron-binding capacity, TIBC) to calculate transferrin saturation (total iron/TIBC). Further investigations are needed to understand the differences between groups in increased plasma iron at 4 weeks in this setting.

Compromised iron status is detrimental to physical performance, and iron supplementation with the intention to increase performance has been used and studied for many years. A meta-analysis, based on data from 22 studies, has shown that iron supplementation in iron-deficient states improves endurance performance and VO_2_max in females in some studies but not in all [24]. In the present study, we did not detect any improvement in VO_2_max or time to exhaustion with ergometer cycling in any group over the 12 weeks. Blood lactate measurement during submaximal workload did not result in any conclusive results. Although blood lactate measured three minutes after maximal workload was higher in the Lp299v group compared to control at weeks 4 and 8, there was no detectable difference in change over time between the groups. Increased blood lactate levels after maximal exhaustion could indicate increased anaerobic capacity. This finding was not associated with any improvement in performance, as measured in our study.

The assessment of self-perceived mood states revealed an improved vigor in the LpFe group compared to control. The increase in vigor score over time correlated positively with the change in ferritin after intake of Lp299v. Our findings were consistent with those observed previously by McClung et al., who found improved vigor in female soldiers supplemented with iron during military training [50], suggesting that improved iron status is the underlying cause of the increased vigor. However, the mechanisms behind this association remain to be explored in future larger studies on athletes with iron deficiency. 

Lp299v has been shown to improve GI wellbeing in a healthy population [51] and in individuals with irritable bowel syndrome [52,53,54], and it has been, therefore, theorized that supplementation with Lp299v might reduce GI side effects during intense exercise and/or following consumption of iron supplements. However, in the present study, no changes or differences in GI wellbeing were observed in any group. According to the reported adverse events, intake of Lp299v together with 20 mg of iron appeared safe and well-tolerated. Future studies should measure the GI wellbeing of the subjects before the start of the study, and a more robust assessment tool should be applied in order to detect possible differences.

URTIs were detected in both groups in the present study, with a lower prevalence in the Lp299v group compared to the control group. Intake of Lp299v has previously been shown to increase the expression of the activation marker CD25 on CD8^+^ T-cells [55]. However, this study was not primarily designed to compare the prevalence, duration, or severity of URTIs, and future studies are needed to properly evaluate this interesting finding. Endurance athletes are more susceptible to URTIs, and elite athletes have a higher rate of URTIs than recreational athletes [56]. Some studies have shown a reduced incidence of URTIs after intake of probiotics, while others have not [57].

The main limitation of this exploratory study was the relatively small sample size, limiting the power and interpretation of data. The URTIs that affected a substantial number of subjects during the intervention weakened the statistical power further, especially at the 8 and 12-week time points.

This was the first study evaluating the effect of a daily intake of Lp299v with a relatively low dose of iron (20 mg) on iron status, physical performance, and mood status in female athletes with low iron stores. Taken together, the results indicated that the intake of Lp299v with 20 mg iron could result in a more substantial and rapid improvement in iron status compared to iron alone. Larger studies are warranted in order to further investigate the effect of Lp299v and iron on iron status as well as the impact on physical performance. Future studies should include elucidation of the mode of action as well as effects on immune functions and mood states.

## Figures and Tables

**Table 1:** Baseline characteristics.

Variables	CtrlFe	LpFe
*n*	23	19
Age (years; SD)	21.6 (6.0)	22.3 (3.5)
Height (cm; SD)	171 (6)	169 (5)
Body weight (kg; SD)	67 (7)	66 (6)
BMI (kg/m^2^; SD)	22.9 (1.8)	23.3 (2.5)
Average training hours per week (SD) *	7.6 (2.0)	8.2 (2.0)
VO_2_max mL × kg^−1^ × min^−1^	2.8 (0.3)	2.9 (0.3)

Data are presented as mean values with standard deviations (SD) for all subjects at the baseline visit. BMI, body mass index. * During the last month prior to the intervention.

**Table 2:** Parameters related to iron status at baseline.

Variables	CtrlFe	LpFe
*n*	22–23	17–18
B-Hb (g/L)	129.4 (9.3)	130.4 (8.7)
P-Ferritin (µg/L)	19.8 (6.8)	19.5 (9.3)
P-Iron (µg/L)	14.7 (6.4)	18.1 (7.5)
P-Transferrin (g/L)	3.2 (0.5)	3.3 (0.5)
P-Transferrin sat. (%)	18.8 (7.9)	22.3 (10.5)
B-EVF (%)	39 (3.0)	39 (2.0)
B-Reticulocytes (pg/L)	46.6 (12.3)	48.5 (11.9)
B-Ret-Hb (pg)	31.3 (2.0)	31.5 (2.4)
sTfR (mg/L)	1.3 (0.3)	1.5 (0.4)
Hepcidin (nmol/L)	1.6 (2.1)	1.9 (2.0)
hCRP (mg/L)	0.9 (0.6)	0.8 (0.3)

Data are presented as mean values with standard deviations (SD) in the per-protocol population. Hb, hemoglobin; EVF, erythrocyte volume fraction; Ret-Hb, reticulocyte hemoglobin content; sTfR, soluble transferrin receptor; hCRP, high-sensitive C-reactive protein.

**Table 3:** Parameters related to iron status. Change from baseline to weeks 4, 8, and 12.

Variable	Week	CtrlFe	LpFe	*p*
B-Hb (g/L)	Week 4	0.55 (8.9)	−1.2 (9.3)	0.7201
	Week 8	2.8 (10.7)	−1.06 (7.2)	0.1876
	Week 12	3.9 (8.7)	0.67 (7.4)	0.5072
P-Ferritin (µg/L)	Week 4	8.2 (7.7)	13.6 (9.9)	0.0565
	Week 8	17.3 (9.6)	25.9 (36.7)	0.9504
	Week 12	17.4 (9.4)	19.5 (19.5)	0.4491
P-Iron (µmol/L)	Week 4	5.6 (10.1)	−1.6 (12.5)	0.0411
	Week 8	2.1 (8.7)	0.81 (11.8)	0.8305
	Week 12	2.6 (6.7)	−0.27 (10.5)	0.4373
P-Transferrin (g/L)	Week 4	−0.16 (0.31)	−0.29 (0.33)	0.3313
	Week 8	−0.31 (0.44)	−0.39 (0.33)	0.8443
	Week 12	−0.18 (0.34)	−0.36 (0.32)	0.1644
P-Transferrin sat. (%)	Week 4	8.2 (12.5)	−1.6 (15.23)	0.0655
	Week 8	4.6 (10.9)	3.56 (15.45)	0.9778
	Week 12	5.1 (10.3)	2.53 (14.67)	0.4056
B-EVF (%)	Week 4	0 (0.03)	0 (0.03)	0.8531
	Week 8	−0.01 (0.02)	−0.01 (0.02)	0.3274
	Week 12	−0.01 (0.02)	−0.01 (0.02)	0.4128
B-Reticulocytes (pg/L)	Week 4	1.4 (9.2)	0.4 (14.0)	0.5148
	Week 8	1.1 (10.9)	−1.3 (11.2)	0.6245
	Week 12	3.5 (9.7)	3.9 (9.3)	0.8014
B-Ret-Hb (pg)	Week 4	1.1 (1.8)	1.0 (1.6)	0.9354
	Week 8	0.8 (1.3)	1.3 (0.5)	0.2749
	Week 12	0.8 (0.9)	1.3 (0.8)	0.0834
sTfR (mg/L)	Week 4	−0.14 (0.20)	−0.12 (0.18)	0.7314
	Week 8	−0.24 (0.27)	−0.15 (0.21)	0.3327
	Week 12	−0.19 (0.15)	−0.18 (0.36)	0.3112
Hepcidin (nmol/L)	Week 4	2.7 (7.3)	0.8 (3.1)	0.3615
	Week 8	1.1 (3.6)	5.9 (8.7)	0.1597
	Week 12	2.5 (4.1)	3.9 (6.8)	0.8076
hCRP (mg/L)	Week 4	−0.05 (0.5)	0.07 (0.4)	0.7876
	Week 8	−0.15 (0.5)	0.01 (0.3)	0.8789
	Week 12	−0.02 (0.6)	0.05 (0.5)	0.9915

Week 4 = change from baseline over the first 4 weeks, Week 8 = change from baseline over the first 8 weeks, Week 12 = change from baseline over the 12-week study; *n* (CtrlFe): ΔV3–V2 = 22–23; ΔV4–V2 = 16–17; ΔV5–V2 = 16–17; *n* (LpFe): ΔV3–V2 = 15–16; ΔV4–V2 = 15–16; ΔV5–V2 = 15.

**Table 4:** Physical performance parameters during maximal workload. Change from baseline to weeks 4, 8, and 12.

Parameter	Change	CtrlFe	LpFe	*p*
Endurance (time to exhaustion, min)	ΔV3–V2	0.29 (0.54)	0.35 (0.35)	0.8888
	ΔV4–V2	−0.17 (1.28)	0.52 (0.78)	0.2428
	ΔV5–V2	−0.30 (1.74)	0.20 (0.69)	0.8467
Heart rate (beats/min)	ΔV3–V2	0.45 (4.56)	0.40 (4.10)	0.9935
	ΔV4–V2	−3.22 (6.50)	−2.00 (2.87)	0.7634
	ΔV5–V2	−2.33 (5.12)	1.29 (4.51)	0.1243
VO_2_max (L/min)	ΔV3–V2	0.07 (0.14)	0.08 (0.13)	0.7357
	ΔV4–V2	−0.08 (0.32)	0.09 (0.18)	0.2863
	ΔV5–V2	−0.03 (0.26)	0.02 (0.14)	0.7717
Lactate (mmol/L)	ΔV3–V2	−0.72 (2.05)	0.47 (1.84)	0.0562
	ΔV4–V2	−1.22 (2.50)	−0.01 (1.42)	0.2743
	ΔV5–V2	−1.05 (2.48)	0.02 (2.06)	0.3346

*n* (CtrlFe: ΔV3–V2 = 19–20; ΔV4–V2 = 8–9; ΔV5–V2 = 14–15; *n* (LpFe): ΔV3–V2 = 14–15; ΔV4–V2 = 10; ΔV5–V2 = 14.
